# Experimental Study on Flexural Behaviour of Prefabricated Steel–Concrete Composite I-Beams Under Negative Bending Moment: Comparative Study

**DOI:** 10.3390/ma18020450

**Published:** 2025-01-19

**Authors:** Huiteng Pei, Shang Zha, Tingying Wu, Baidian Li, Gangyi Zhan, Wenqin Deng

**Affiliations:** 1 Jiangxi Communication Design and Research Institute Co., Ltd., Nanchang 330052, China; 2180098@tongji.edu.cn (H.P.);; 2 School of Civil Engineering, Nanjing Tech University, Nanjing 211816, China; 3 Jiangxi Provincial Communications Investment Group, Ltd., Nanchang 330108, China; 4 China Railway Shanghai Design Institute Group Co., Ltd., Shanghai 200070, China

**Keywords:** steel–concrete composite I-beams, negative bending moment zone, ultra-high-performance concrete, corrugated steel webs, crack resistance, ultimate bearing capacity

## Abstract

The issues of numerous steel beam components and the tendency for deck cracking under negative bending moment zones have long been challenges faced by traditional composite I-beams with flat steel webs. This study introduces an optimized approach by modifying the structural design and material selection, specifically substituting flat steel webs with corrugated steel webs and using ultra-high-performance concrete for the deck in the negative bending moment zone. Three sets of model tests were conducted to compare and investigate the influence of deck material and web forms on the bending and crack resistance of steel–concrete composite I-beams under a negative bending moment zone. The findings indicate that, compared to a conventional steel–normal concrete composite I-beam, incorporating ultra-high performance concrete into the negative bending zone enhances the cracking load by 98%, resulting in finer and denser cracks, and improves the ultimate bearing capacity by approximately 10%. In comparison to the composite I-beam with flat steel webs, the longitudinal stiffness of the composite I-beam with corrugated steel webs is smaller, which can further enhance the bridge deck’s resistance to cracking in the negative bending moment zone, and maximize the steel-strengthening effect of the lower flange of the steel I-beam. Based on the findings of this study, it is recommended to use steel ultra-high-performance concrete composite I-beams with corrugated steel webs due to their superior crack resistance, bending strength, and efficient material utilization.

## 1. Introduction

Steel–normal concrete (NC) composite I-beam bridges are widely used in bridge construction due to advantages such as reduced weight, material reusability, and rapid construction. However, issues like stiffener requirements, extensive welding, fatigue effects, limited structural efficiency, and cracking under significant negative bending moments in steel–NC composite I-beams have raised concerns about stiffness degradation and durability [1,2,3].

**Figure 1:**
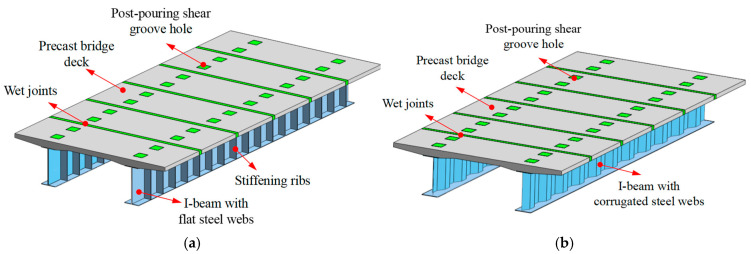
Schematic diagram of steel–concrete composite I-beams: (**a**) composite I-beam with FSWs; (**b**) composite I-beam with CSWs.

Corrugated steel webs (CSWs), known for their excellent buckling resistance, eliminate the need for vertical and horizontal stiffeners, enabling standardized factory production and reducing welding workload [4,5,6,7,8]. Composite I-beams are more practical for medium- and small-span bridges, leveraging the compressive strength of concrete and tensile strength of steel for high material efficiency. On-site shear connector construction also simplifies assembly [9]. This paper proposes replacing flat steel webs (FSWs) in traditional steel–NC composite I-beams (Figure 1a) with CSWs (Figure 1b) to enhance structural efficiency by reducing welding and increasing transverse stiffness, thereby improving industrial production. Furthermore, ultra-high-performance concrete (UHPC), with its superior mechanical properties and durability, addresses cracking issues in the negative bending moment region and extends the material’s structural lifespan [10,11,12,13,14]. The combination of CSWs and UHPC is expected to address key technical challenges in prefabricated steel–NC composite I-beam bridges, improving the performance of composite I-beam bridges [15,16,17].

The cracking issue of the concrete deck in the negative bending moment zone remains a critical technical challenge, impacting both the durability and the efficient construction of steel–NC composite I-beam bridges. To address this issue, many scholars have reduced the risk of concrete cracking under the negative bending moment zone of composite I-beams through structural design optimization. Nie et al. [18] discovered that factors such as the force ratio, reinforcement ratio, and shear connector spacing significantly affected the crack width in the negative bending moment region of composite I-beams. They also developed a formula to calculate the average crack spacing and width based on model tests. Manfredi et al. [19] established a refined stress model for the negative bending moment region of composite I-beams based on the slip of steel beams and concrete slabs and the slip of rebars and concrete. Men et al. [20] investigated how parameters like reinforcement ratio, concrete slab thickness, and web thickness influence the structural performance of steel–NC composite I-beams under flexural shear coupling in the negative bending moment region, using both model tests and numerical simulations. Lee et al. [21] investigated the impact of high-strength steel on the flexural ductility of continuous composite I-beam bridges under the negative bending moment, highlighting that as steel strength increases, the plastic deformation capacity of steel–NC composite I-beams diminishes, leading to a substantial reduction in beam flexural rigidity. Song et al. [22] carried out a fatigue test study in the negative bending moment zone of steel–NC composite I-beams, and put forward suggestions for the fatigue design of steel–NC composite I-beams under the action of the negative moment. Considering material and structural nonlinearity, Zhou et al. [23] investigated the distortion instability of steel–NC composite I-beams subjected to negative bending moments, and provided practical formulas for calculating the slenderness ratio and ultimate bending moment stability coefficient. Additionally, some scholars have explored construction measures to enhance the crack resistance of the deck under negative bending moments. Sun et al. [24] and Fan et al. [25] are conducting ongoing studies on the impact of prestressing technology, the pull-out and non-shear connection methods, and fiber-reinforced cement-based composites on crack resistance under negative bending moments in steel–concrete composite I-beam bridges, and proposed corresponding formulas for calculating bending crack widths. Liu et al. [26] studied the reinforcement effect of longitudinal reinforcement on the negative bending moment zone of continuous-span prestressed concrete beam bridges, which provided technical support for the reinforcement design of concrete beam bridges during their service period. Wang et al. [27] designed eight test beams, studied the influence of shear joints, prestress, deck width, and deck construction technology on the crack resistance and bending performance of steel–NC composite I-beams in the negative bending moment zone, and proposed the correction calculation method of crack spacing and width under the negative bending moment zone. In addition to the aforementioned measures, some scholars have found that optimizing the material properties of the bridge deck can also alleviate, to a certain extent, the issue of cracking under the negative bending moment zone. Qi et al. [28] pointed out that using UHPC can improve the stiffness and crack resistance of steel–concrete composite I-beams under the negative bending moment zone, and proposed a simplified formula for calculating the crack width and flexural bearing capacity of steel–UHPC composite I-beams. Hamoda et al. [29] investigated the impact of different concrete types (NC, steel fiber-reinforced concrete (SFRC), and ECC) on the mechanical behavior of steel–concrete composite I-beams subjected to negative bending moments. They highlighted that using high-performance concrete enhances crack resistance in regions experiencing negative bending. Zhang et al. [30] and Qi et al. [31] performed bending tests on steel–UHPC composite I-beams in the negative bending moment region. Their findings indicated that, in comparison to NC, UHPC bridge decks formed more numerous and closely spaced cracks, leading to a notable increase in the cracking load of the composite I-beams. Lu et al. [32] utilized UHPC-NC deck panels in the negative bending moment zone of composite I-beams, which improved their bending performance and simultaneously reduced the amount of UHPC required. Wu et al. [5] reported that the bending capacity of composite I-beams using UHPC in the negative bending moment zone was 17.9% higher than that of those using NC.

The above-mentioned studies primarily focus on improving the crack resistance of composite I-beams in the negative bending moment region by optimizing structural parameters or materials with FSWs. However, the structure proposed in this paper suggests replacing FSWs with CSWs and employing UHPC for the deck panel. Compared to FSWs, CSWs possess unique corrugated structural characteristics and superior local stability. However, this also results in lower longitudinal stiffness, which may lead to differences in the structural performance of composite I-beams with CSWs compared to those with FSWs. However, existing research has mainly focused on composite I-beams with FSWs, and there is limited research on the negative bending moment structural properties of composite I-beams with CSWs. Furthermore, the effects of combining corrugated steel web I-beams with UHPC deck panels remain unknown. Therefore, in this study, three sets of test beams were first designed and fabricated, incorporating different web configurations (CSWs and FSWs) and deck materials (NC and UHPC). Single-point loading tests were then conducted to evaluate the structural performance of the test beams under negative bending moments, with a focus on cracking, bending strength, and stiffness. Finally, a comparative analysis was performed to examine the influence of different web configurations and deck materials on the crack resistance and bending performance of the composite beams.

## 2. Experimental Program

### 2.1. Specimen Design

**Figure 2:**
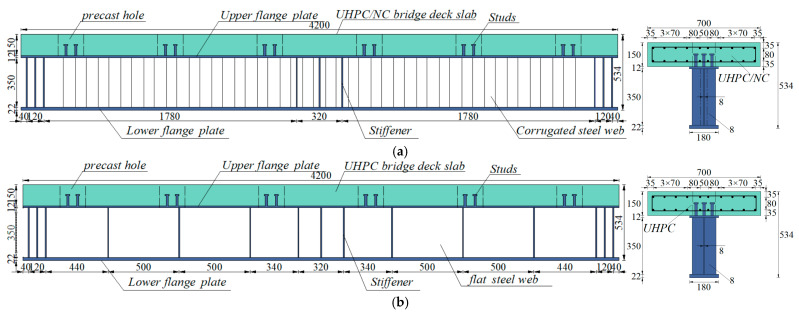
Dimensions and details of specimens (mm): (**a**) A1/A3; (**b**) A2.

**Figure 3:**
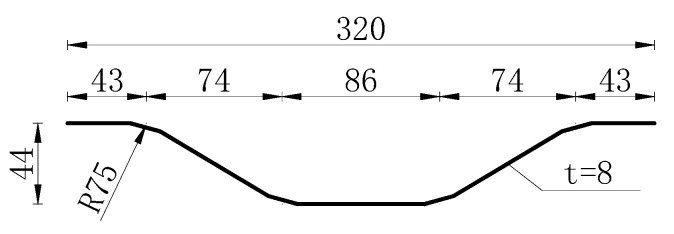
Dimensional size of CSWs (mm).

To analyze the impact of web shape and deck strength on the composite beam, three sets of test specimens (A1–A3) were designed at a 1:10 scale based on the main span length of an actual 25 + 45 + 25 m bridge. The cross-sectional design ensured that the relative position of the neutral axis remained consistent with that of the actual bridge’s cross-section, ensuring that the stress distribution in the test beams closely resembled that of the actual bridge. Test specimens A1 and A2 utilized CSWs and FSWs as the steel beam webs, respectively, with UHPC material used for the concrete deck. Apart from the difference in web form, all other dimensional parameters of the specimens remained the same. Similarly, test specimens A1 and A3 both employed CSWs as the steel beam webs, but with UHPC and NC materials used for the concrete deck, respectively, and the dimensional parameters of the specimens were identical. Loading tests were conducted on A1 and A2 to evaluate the effect of web configuration, and on A1 and A3 to assess the impact of deck material on the structural performance of composite I-beams in the negative bending moment zone. The structure of the specimens is shown in Figure 2. The total length of each specimen was 4.2 m, with a 4.0 m spacing between the supports. The height of the composite I-beams was 534 mm, and the width and thickness of the concrete deck were 700 mm and 150 mm, respectively. The concrete deck contained two layers of longitudinal rebar spaced 80 mm apart, with a transverse spacing of approximately 70 mm. In areas with shear studs, the spacing was slightly adjusted, as shown in Figure 2. Additionally, stirrups were arranged at 90 mm intervals along the longitudinal direction of the deck. All rebar had a diameter of 12 mm. The height of the steel beams was 384 mm. Specifically, the width and thickness of the upper flanges on the steel beams were 180 mm and 12 mm, respectively. The lower flanges had a width and thickness of 180 mm and 22 mm, and the height and thickness of the web were 350 mm and 8 mm, respectively. The size of the reserved shear holes was 180 × 180 mm, and the spacing between the holes was 700 mm. The three sets of composite I-beams were equipped with transverse stiffeners at the support and loading positions, with a thickness of 8 mm. Additionally, to enhance the stability of the flat steel web, specimen A2 was further reinforced with stiffeners spaced approximately 500 mm apart. The steel I-beams were made of Q355 steel, and cluster studs were arranged on the upper flange of the steel I-beams. Each specimen was designed with six groups of clustered studs, with 12 studs per group. The center spacing of the shear slots where the clustered studs were located was 700 mm. The transverse and longitudinal spacing of the studs were 40 mm and 70 mm, respectively, with a stud size of Φ16 × 90, made from ML15AL material. The arrangement of the studs conformed to the relevant provisions of GB 50917-2013, ensuring an adequate number of studs to connect the concrete deck and the steel beam [33]. The dimension of the CSW is shown in Figure 3, and the thickness of the CSWs was consistent with that of the FSWs. The specimen parameters are shown in Table 1.

### 2.2. Material Properties

Prior to the experimental loading, three sets of samples were prepared for each material to conduct material property tests. For NC, material property tests were conducted based on C50-grade concrete provided by the factory. For UHPC, the mix design was as follows: cement/silica fume/fly ash/quartz powder/quartz sand/water/superplasticizer = 1.00: 0.25: 0.10: 0.25: 1.10: 0.24: 0.027. In addition, material property tests were performed after the mix design was completed. While casting the bridge deck concrete, three groups of 150 × 150 × 150 mm cubic specimens of NC and UHPC were prepared simultaneously, along with three groups of UHPC dog-bone specimens. The compressive strength of NC and UHPC after 28 days of curing, as well as the tensile strength of UHPC, were measured following the guidelines outlined in GB/T 50081–2019 and T/CECS 10107-2020 [34,35]. The steel beam components were fabricated from Q355 steel, and the reinforcing bars were constructed using HRB400 rebars, both sourced from the batch used for the test specimens. The tensile properties of both materials were tested in accordance with GB/T 228.1-2021 [36]. The average values of all components were taken as the experimental results, and the material performance parameters are shown in Table 2. The elastic moduli of NC and UHPC are 34,500 MPa and 45,200 MPa, respectively. The elastic modulus of steel and rebar is 206,000 MPa. Based on the yield strengths of steel and rebar listed in Table 2, their corresponding yield strains were calculated to be 1868 με and 2175 με, respectively. In the following discussion, when the strain of steel or rebar reaches these values, they are considered to have yielded.

### 2.3. Specimen Fabrication and Processing

**Figure 4:**
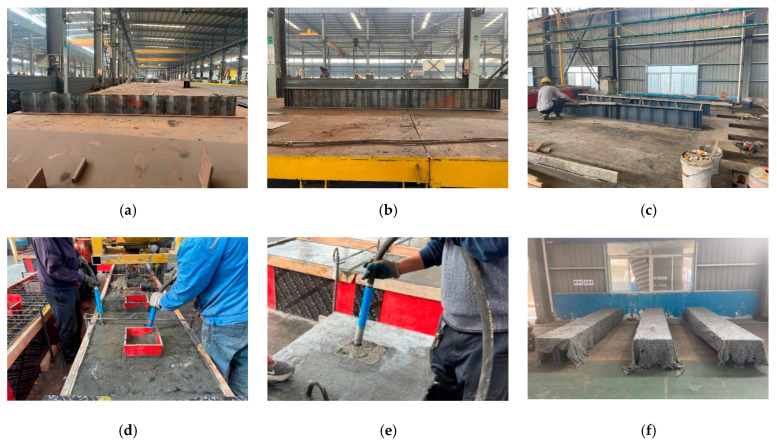
Specimen preparation: (**a**) CSW processing; (**b**) steel I-beam with CSW processing; (**c**) steel I-beam with FSW processing; (**d**) concrete was poured and vibrated in the areas outside the slots where the stud groups were located; (**e**) concrete was poured and vibrated in the slots where the stud groups were located; (**f**) maintenance.

Due to the need for high-temperature curing of UHPC deck panels in the negative bending moment zone, using a cast-in-place method on-site would delay construction and increase the difficulty of the work. Therefore, using precast UHPC deck panels for the assembly construction in the negative bending moment zone can reduce the amount of on-site cast-in-place concrete, ensuring better quality control. In this study, the steel beams of the test specimens were processed and assembled in the factory, while the concrete deck panels were cast in two batches to simulate an assembly construction method. During the first pouring stage, concrete was poured from top to bottom outside the area where the stud groups were located, and wooden formwork was used to separate the areas with stud groups. Once the prefabricated concrete deck formed from the first pouring stage attained a certain strength, the concrete decks were placed on top of the steel beams, with the studs of the steel beams inserted into the precast holes of the concrete decks. Finally, concrete was poured into the precast holes, and the concrete around the studs was vibrated repeatedly to ensure compaction. Subsequently, the surface was smoothed out and cured. This construction sequence was designed to replicate the stages involved in the prefabricated assembly of a bridge. The fabrication process is illustrated in Figure 4.

### 2.4. Measuring Point Layout and Loading Scheme

**Figure 5:**
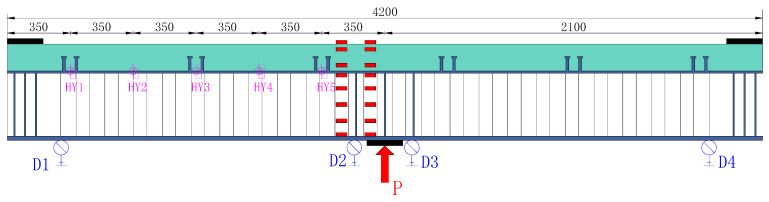
Measurement scheme (mm).

**Figure 6:**
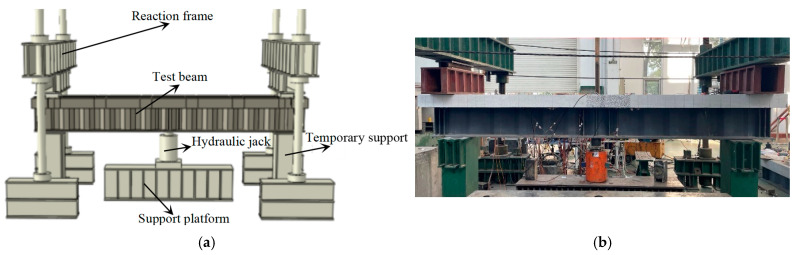
Test loading: (**a**) loading model; (**b**) experimental load application at the site.

The test contents mainly included the load-vertical deflection relation, relative slip of the steel I-beam and concrete slab, concrete slab strain, crack width and spacing, rebar strain in the concrete slab, steel beam roof and bottom plate strain, web plate strain, etc. The strain measuring points were arranged on the concrete deck, rebar, and steel I-beam, especially in the mid-span region, with a denser arrangement, as shown in Figure 5. In Figure 5, D1–D4 refer to four displacement transducers placed on the bottom of the beam to measure the deflection at various points along the longitudinal axis of the beam. HY1–HY5 refer to displacement transducers located beneath the concrete deck to measure slip between the steel and concrete components. In order to facilitate the observation of deck cracking, the jack was placed at the mid-span below the specimen, and the specimen was constrained by a reaction frame. Before the formal test, a preload of 20 kN was applied to eliminate non-elastic deformations in the equipment and supports. Subsequently, the test commenced with an initial load of 0 kN, and the specimens were loaded in 50 kN increments. Each load level was held for 2 min to collect data from sensors and record the load at which the primary crack appeared, as well as the crack width and propagation. The specimens were initially subjected to load-controlled incremental loading until the applied force could no longer increase. At this point, the loading protocol shifted to displacement control, with increments of 2 mm per step, until the specimens were completely destroyed. The loading setup is illustrated in Figure 6.

## 3. Experimental Results and Discussion

### 3.1. Failure Mode

**Figure 7:**
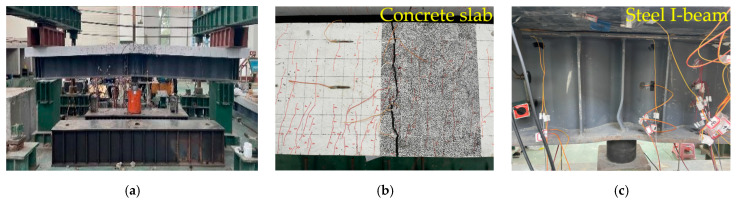
Failure mode of specimen A1: (**a**) overall deformation; (**b**) cracking of the concrete slab; (**c**) steel I-beam buckling.

**Figure 8:**
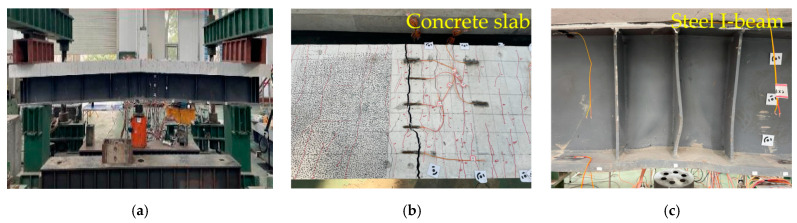
Failure mode of specimen A2: (**a**) overall deformation; (**b**) cracking of the concrete slab; (**c**) steel I-beam buckling.

**Figure 9:**
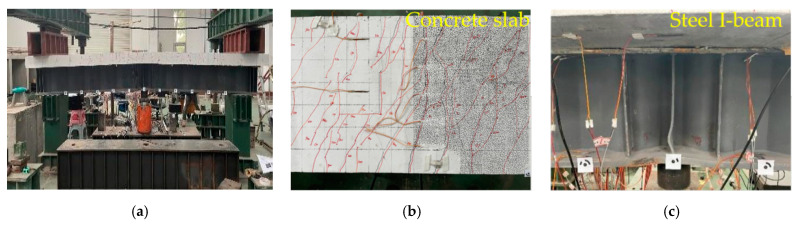
Failure mode of specimen A3: (**a**) overall deformation; (**b**) cracking of the concrete slab; (**c**) steel I-beam buckling.

As shown in Figure 7, Figure 8 and Figure 9, the cracking load of specimen A1’s concrete slab was 249 kN, with the first crack occurring at the mid-span and near the two slots along the span. When the load reached 300 kN, the major cracks near the mid-span slot extended through the concrete slab. At a load of approximately 1000 kN, local buckling occurred in the lower flange of the steel beam near the loading point. Specimen A2 showed that the cracking load of the concrete slab was 210 kN, and the cracks first appeared at the bottom of the mid-span. When the loading force reached 300 kN, the main crack was near the mid-span slot and the mid-span penetrated the concrete slab. When the loading force reached about 1000 kN, local compression buckling occurred near the loading point. In the case of specimen A3, the cracking load of the concrete slab was 124 kN, with initial cracks emerging at the junction between the reserved shear slot and the deck. When the applied loading force reached 190 kN, both the slot near the mid-span and the main crack at the mid-span penetrated the concrete slab. When the applied load reached 980 kN, both the lower flange of the steel beam close to the loading point and the CSWs near the bottom plate experienced local buckling.

In summary, all three specimens exhibited typical flexural failure characteristics, including bending-induced cracking of the bridge deck and local buckling of the steel beam’s bottom flange. As the load increased, cracks first appeared at the shear slot in the span, and then, through-cracks appeared in the middle span. The longitudinal tensile rebars yielded on the deck, and then, the upper flange of the steel I-beam was strained and yielded. With the increase in crack width, the lower flange and web of the steel I-beam appeared to buckle locally in compression, and the loading terminated. It is worth noting that, compared to specimen A2, the cracking development of specimen A1 was slower and distributed more evenly. In contrast to the wide and extensive cracks observed in specimen A3 with an NC bridge deck, the cracks in specimen A1 with a UHPC bridge deck were finer and denser.

### 3.2. Load–Displacement Curve

**Figure 10:**
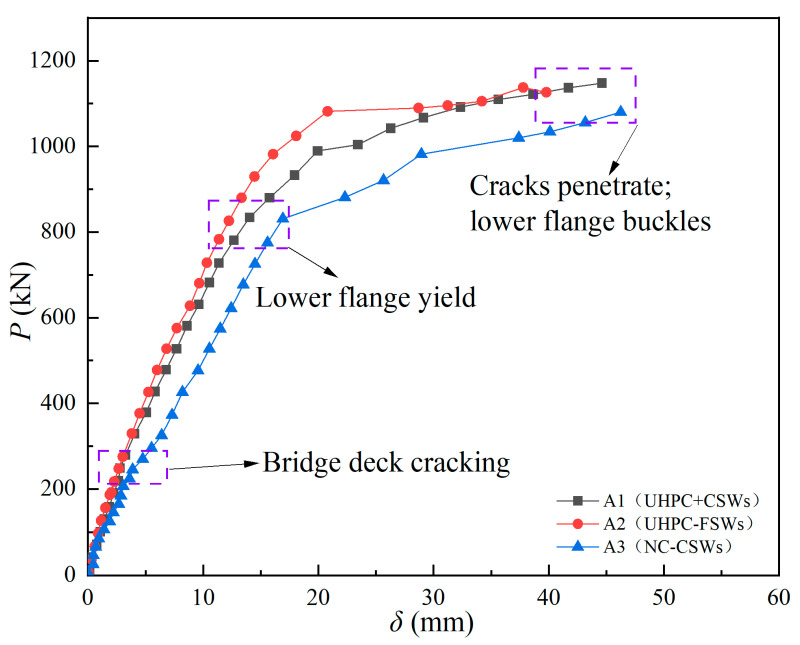
Load–displacement curves.

Figure 10 presents the load–displacement curves for the three specimens under loading, with the key test results provided in Table 3. In the table, *P*_cr_ and *δ*_cr_ represent the cracking load and the corresponding deflection value, *P*_y_ and *δ*_y_ represent the yield load of the steel beam and the corresponding deflection value, and *P*_u_ and *δ*_u_ represent the ultimate load of the steel beam and the corresponding deflection value. *K*_1_ and *K*_2_ represent the initial stiffness and elastic stiffness of the specimen, respectively, determined as the ratio of the corresponding load to displacement. D = *δ*_u_/*δ*_y_ is the ductility ratio of the specimen. As shown in Figure 10, the load–displacement curves of the three specimens can be roughly divided into four stages: the linear elastic stage (I) (no cracks), the cracking stage (II) (crack propagation), the yield stage (III) (crack penetration to steel beam yield) and the strengthening stage (Ⅳ) (steel strengthening to local buckling). Linear elasticity stage (I): the deck concrete did not crack, and the steel–concrete composite I-beams worked together. Cracking Stage (II): there was a certain difference between A1/A2 (steel–UHPC composite I-beam) and A3 (steel–NC composite I-beam) regarding the cracking of the deck concrete and the penetration of the crack. Because of the superior tensile strength of UHPC, it could still provide a certain tensile contribution even after the cracking of the deck, resulting in only a marginal reduction in the stiffness of A1/A2 specimens. At this stage, the load of A1/A2 was jointly borne by the UHPC deck, rebars and steel beams. However, the NC deck used in A3 had low tensile strength, so the concrete slab failed after cracking. Therefore, the slope of the load–displacement curve of A3 decreased significantly in the cracking stage (II), and the load of A3 was jointly borne by the rebar and steel beam at this stage. Yield stage (III): with the increase in load, the displacement increased rapidly, and the rebars of the deck entered the strengthening phase, while the upper and lower flanges of the steel I-beam reached the yield strength. Strengthening stage (IV): the steel entered the strengthening stage, with the crack width rapidly widening. Although the load stayed nearly the same, there was a significant increase in the mid-span deflection, which was subsequently followed by local buckling of the compression flange plates and webs.

The cracking loads, *P*_cr_ and *P*_u_, of specimen A1 were 18.6% and 1.9% higher than that of specimen A2, and the ductility ratio of *D* was also 31.5% higher, as shown in Table 3. The yield load *P*_y_ and stiffness (*K*_1_ and *K*_2_) decreased by 17.4%, 2.3%, and 3.2% in specimen A1, respectively. Within the region of the negative bending moment, tensile stress on the bridge deck is primarily caused by the loading of the steel beams. The stud connections restrain the longitudinal sliding in the interface between the bridge deck and the steel beams, resulting in tensile stress within the bridge deck. When the lower flange of the steel beam is under compression in negative bending, the CSW exhibits weak longitudinal stiffness due to its accordion effect, providing a certain longitudinal expansion and contraction space. This diminishes the tensile stress transferred from the steel beams to the bridge deck through the connections, which, in turn, reduces the likelihood of cracking in the bridge deck. It can be seen that the composite I-beam with CSWs had better cracking resistance and ductility than the composite I-beam with FSWs. The difference in flexural bearing capacity between the two beams was small, and the yield strength and flexural stiffness were slightly reduced. Compared with specimen A3, the *P*_cr_, *P*_u_, *K*_1_, *K*_2_, and *D* of specimen A1 increased by 100.8%, 5.7%, 43.4%, 29.3%, and 32.4%, respectively, while the yield strength *P*_y_ decreased by 5.6%. Due to the high strength of UHPC, the neutral axis position in specimen A1 was higher, which increased the distance to the bottom flange of the steel beam, leading to earlier yielding. The cracking load of the deck using UHPC was doubled, and the stiffness and ductility were also greatly improved, but the influence on flexural capacity was small.

### 3.3. Analysis of Crack Characteristics of Deck

**Figure 11:**
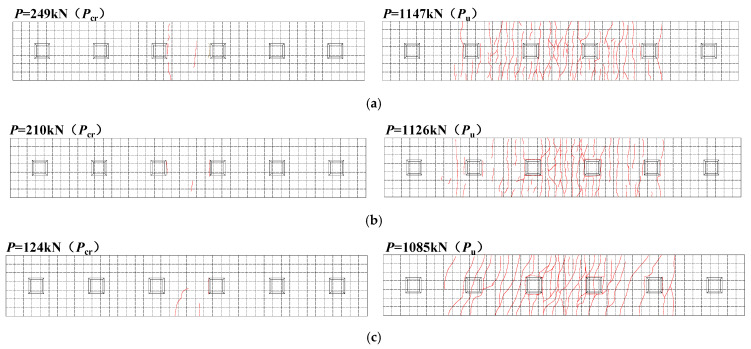
Crack development diagram of specimens: (**a**) A1; (**b**) A2; (**c**) A3.

Figure 11 illustrates the crack distribution in the specimens. The grid in the figure is a 100 mm × 100 mm reference mesh to aid in the identification of crack positions, and the red line represents the crack. When compared to specimen A2, specimen A1 exhibited an 18.6% increase in the cracking load, with the first cracks appearing at the junction between the mid-span shear slot and the interface of the old and new concrete in the prefabricated deck. As a result of the accordion-like deformation of the CSWs, its longitudinal stiffness is relatively weak, thereby reducing the restraint of the steel beam on the bridge deck and effectively preventing excessive stress concentration within the bridge deck. This resulted in the slow development of the specimen A1 cracks and the more uniform distribution of cracks. The cracking load of specimen A1 exceeded that of specimen A3 by 100.8%. The cracks in the UHPC slab were fine and dense, while the cracks in the NC slab were wide and thick.

**Figure 12:**
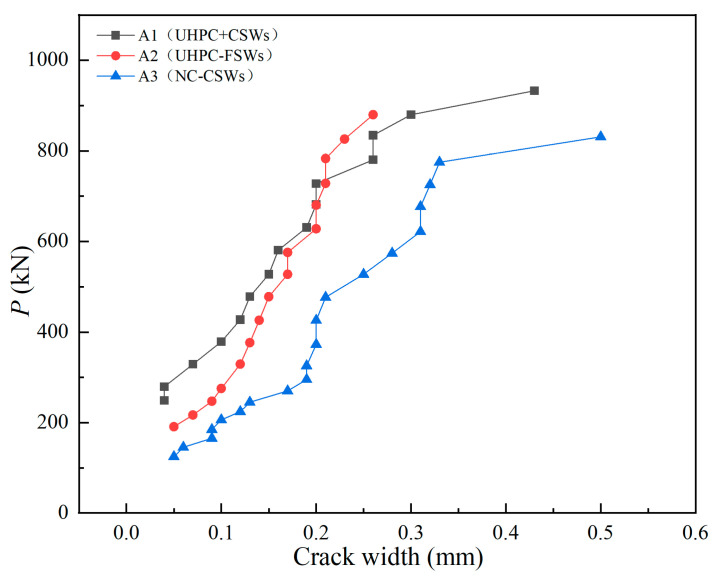
Load–maximum crack width curves of specimens.

Figure 12 illustrates the curves of load versus maximum crack width for each test beam. The maximum crack width expansion rate of the three beams was A1 < A2 < A3. Because of the excellent tensile strength of UHPC and the accordion effect of the CSWs, specimen A1 became the composite I-beam with the best tensile performance. For steel–UHPC (A1/A2) composite I-beams, when the width was less than 0.2 mm, the maximum crack width expansion rate increased slightly with the increase in load. As the ultimate load approached, the main crack width increased sharply with the slow increase in load. The crack expansion rate of the steel–NC composite I-beam (A3) was larger from the beginning. It is worth noting that the crack widths of the two groups of composite I-beams with CSWs increased greatly at around 720 kN. This occurrence was attributed to the mid-span sections of the lower flange in both groups reaching the yield strain at 720 kN. Subsequently, the displacement increased significantly with increasing load, resulting in substantial widening of the crack width.

### 3.4. Strain Distribution of Steel I-Beams

**Figure 13:**
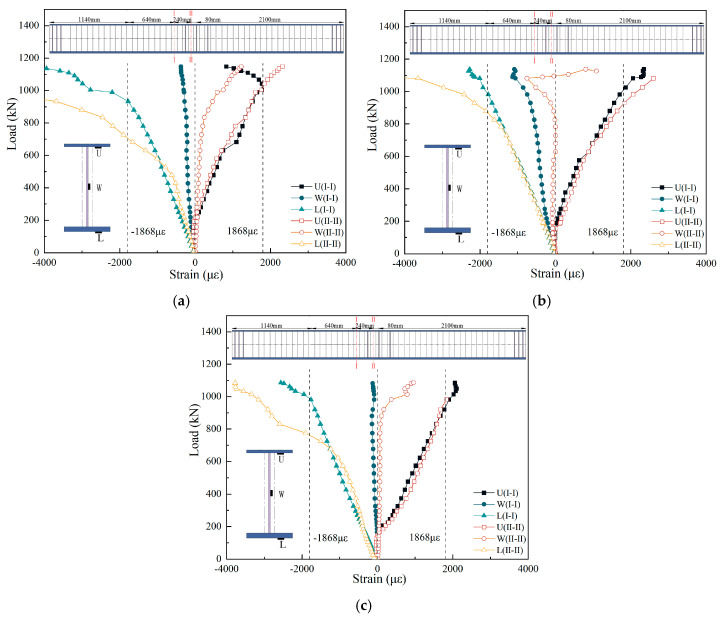
Load–strain curves of steel I-beams: (**a**) A1; (**b**) A2; (**c**) A3.

Figure 13 presents the load–strain curves of the steel I-beams. The strain behavior of the steel I-beams in all three specimens was observed to be quite similar. The strains of the upper flange, web, and lower flange of the steel I-beams all increased with the increase in load, but the increase in amplitude was different. For A1 and A3, the strain growth rate in the lower flange was the highest, followed by the upper flange, with the web being the lowest; for A2, the strain growth rates of the lower and upper flanges were similar, while the web remained the lowest. The yield load points of the lower flange in the steel I-beams of specimens A1, A2, and A3 were 727 kN, 880 kN, and 770 kN, respectively. It can be seen that the yield load of steel I-beams of the composite I-beams with CSWs (A1 and A3) was lower than that of composite I-beams with FSWs (A2). This difference was primarily attributed to the weaker longitudinal stiffness of steel I-beams with CSWs. The center axis of the composite section was positioned higher compared to that of the steel I-beam with FSWs, causing the lower flange of the steel I-beam with CSWs to reach the yield stage earlier than that with FSWs. For the I-I section near the mid-span, it can be seen that the web strain of composite I-beams with CSWs increased only slightly with the increase in load, while the web strain of composite I-beams with FSWs increased to a greater extent with the increase in load. This phenomenon was attributed to the folding effect of the CSWs, where the corrugation of the web diminished the capacity to withstand axial forces in the longitudinal direction, consequently facilitating strain release along the longitudinal axis. In addition, by comparing the strain distribution of specimen A1 and specimen A2, it can be seen that the compression strain of the lower flange plate in the middle span of specimen A1 and near specimen A1 entered the strengthening stage. The tensile strain was significantly greater than that of specimen A2, which also indicated that the bending contribution of the lower flange of specimen A1 was higher than that of specimen A2. Consequently, even when disregarding the bending resistance of the CSWs, the bending capacity in the negative bending moment zone of the composite I-beam with CSWs remained comparable to that of the composite I-beam with FSWs.

### 3.5. Strain Variation Across the Height of the Beam

**Figure 14:**
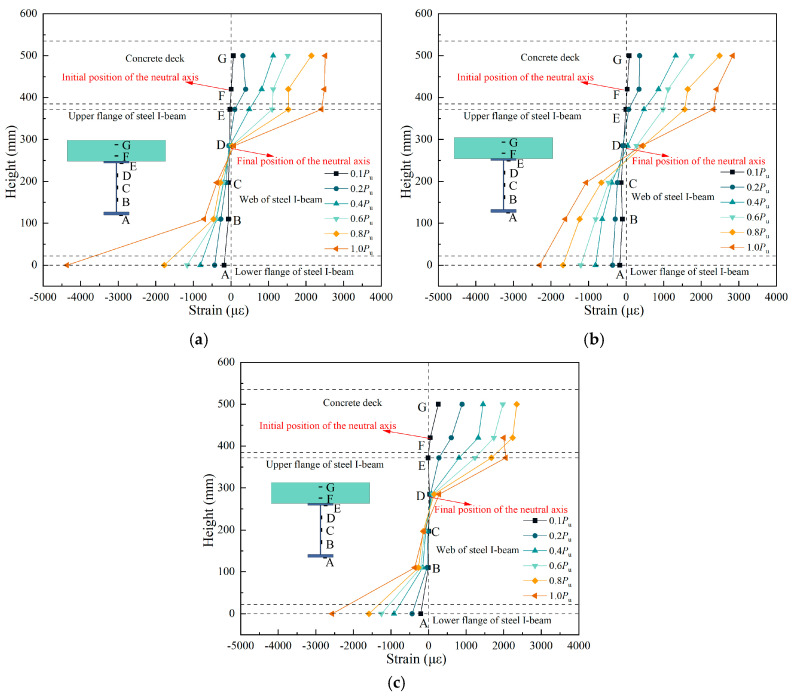
Strain distribution along beam height: (**a**) A1; (**b**) A2; (**c**) A3.

Figure 14 illustrates how the normal strain varies with the height of the beams in the three specimens. A–G represent the strain measurement points arranged at key positions along the section. It can be seen that compared with specimen A2, the composite I-beams with CSWs (specimen A1 and specimen A3) were affected by the accordion effect, and the normal strain of CSWs was smaller. During the initial loading stage (0.1 *P*_u_), the neutral axis of all three specimens was located within the concrete slab. As the load increased and the concrete slab began to crack, the neutral axis shifted downward. When the neutral axis moved below the upper flange, the upper flange transitioned from compression to tension, reaching its yield strength at the ultimate load stage (1.0 *P*_u_). As shown in Figure 14b, the longitudinal strain distribution of specimen A2 conforms to the plane section assumption, meaning that the longitudinal strains of the concrete slab, the upper flange, the web, and the lower flange of the steel beam vary approximately linearly along the height of the cross-section. In comparison, the strain in the CSWs of specimens A1 and A3 is significantly smaller, with only the concrete slab, upper flange, and lower flange of the steel beam conforming to the plane section assumption. Upon reaching the ultimate load *P*_u_, the compressive strain of the lower flange of specimen A1 was 4480 με, while the maximum compressive strains on the compression side of specimen A2 and specimen A3 were 2400 με and 2420 με, respectively. In other words, considering the combined effect of CSWs and UHPC, the compressive strain-strengthening effect of specimen A1’s lower flange was more significant, which improved the bending capacity of the composite I-beam under the negative bending moment zone. This made up for the shortcomings of weak axial stiffness and small flexural contribution of CSWs.

### 3.6. Relative Slip Between Steel I-Beam and Concrete Deck

**Figure 15:**
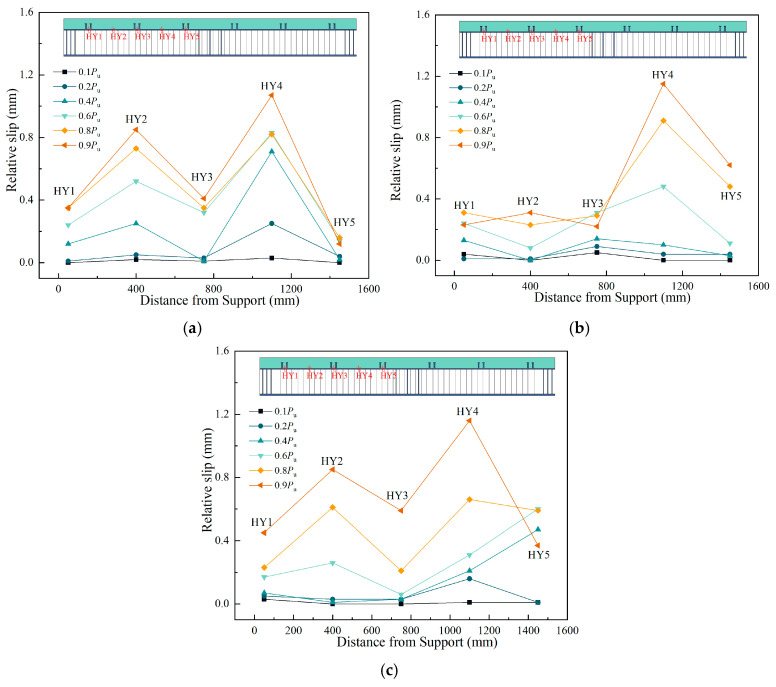
Relative slip along the beam length: (**a**) A1; (**b**) A2; (**c**) A3.

Figure 15 shows the relative slip between the steel I-beam and concrete deck along the beam length of the three specimens. The results show that, due to the group stud effect, the maximum slip along the beam length of the three specimens occurred at the HY4 position, while the slip at the shear slot location was smaller compared to areas without shear studs. The maximum slip of the three sets of specimens did not exceed 1.2 mm, with the composite I-beams using UHPC bridge decks exhibiting slightly less slip than those using NC bridge decks.

## 4. Conclusions

This study explores the impact of web form and concrete deck strength on the Structural properties of composite I-beams in the negative bending moment zone through loading tests on three steel–concrete composite I-beams. The key findings are as follows:

(1) Each of the three specimens exhibited typical bending failure characteristics, with a similar failure process. Initially, cracks developed at the mid-span shear slot, followed by the formation of through-cracks at the mid-span. Subsequently, the longitudinal tensile rebar on the deck yielded, and then, the upper flange of the steel I-beam experienced tensile yielding. With the increase in the width of the crack, the lower flange and web of the steel I-beam appeared to undergo local compression buckling, leading to the failure of the specimen.
(2) Compared to the steel–UHPC composite I-beam with FSWs, the steel–UHPC composite I-beam with CSWs demonstrated reduced longitudinal stiffness, which can be attributed to the accordion-like behavior of the web. This allowed for longitudinal expansion and contraction, reducing the tensile stress transmitted from the bottom of the steel beam to the bridge deck within the negative bending moment zone. As a result, they demonstrated superior crack resistance and ductility, with the cracking load increased by approximately 19%.
(3) In comparison to the steel–NC composite I-beam with CSWs, the steel–UHPC composite I-beam with CSWs demonstrated twice the cracking load, as well as a notable increase of more than 30% in both stiffness and ductility. The cracks in the UHPC deck were fine and dense, while those in the NC bridge deck were wide and extensive. Additionally, UHPC has a certain effect on reducing the slip between steel and concrete.
(4) The combination of UHPC and CSWs contributed to a more significant steel-strengthening effect in the plastic phase of the steel I-beam’s lower flange compared to the steel I-beam with FSWs. This strengthening effect in the steel material contributed to improving the bending performance of the steel–UHPC composite I-beam with CSWs under negative bending moments, thereby compensating for the reduced flexural contribution caused by the lower longitudinal stiffness of the CSWs. Thus, the steel–UHPC composite I-beam with CSWs not only exhibited excellent crack resistance, but also fully utilized the characteristics of the materials. In the future, the UHPC deck could be further optimized to enhance its economic efficiency, promoting the wider application of this structure in practical engineering projects.

## Figures and Tables

**Table 1:** Design parameters of specimens.

No.	Web Type	Deck Material
A1	CSWs	UHPC
A2	FSWs	UHPC
A3	CSWs	NC

**Table 2:** An overview of the mechanical properties of the specimens.

NO.	NC		UHPC		Steel Plates		Rebars	
	*f*_ck,cube_/MPa	*f*_t_/MPa	*f*_ck,cube_/MPa	*f*_t_/MPa	*f*_y_/MPa	*f*_u_/MPa	*f*_ry_/MPa	*f*_ru_/MPa
1	61	2.3	147	6.3	385	535	441	605
2	65	2.1	145	6.8	389	539	457	626
3	57	2.5	133	6.4	382	546	448	613
Average	61	2.3	142	6.5	385	540	448	614

Note: *f*_ck,cube_ is the concrete cube compressive strength, *f*_t_ is the axial tensile strength of NC and UHPC, *f*_y_ is the yield strength of steel, *f*_u_ represents the ultimate strength of steel, *f*_ry_ is the yield strength of rebars, *f*_ru_ is the ultimate strength of rebars.

**Table 3:** Test results.

No.	*P*_cr_/kN	*δ*_cr_/mm	*K*_1_/(kN·mm^−1^)	*P*_y_/kN	*δ*_y_/mm	*K*_2_/(kN·mm^−1^)	*P*_u_/kN	*δ*_u_/mm	*D*
A1	249	2.79	89.25	727	11.39	63.83	1147	44.63	3.92
A2	210	2.29	91.70	880	13.34	65.96	1126	39.80	2.98
A3	124	1.90	65.26	770	15.60	49.36	1085	46.25	2.96

## Data Availability

The original contributions presented in this study are included in the article. Further inquiries can be directed to the corresponding author.
